# Development and validation of novel nomograms for predicting the survival of patients after surgical resection of pancreatic ductal adenocarcinoma

**DOI:** 10.1002/cam4.2959

**Published:** 2020-03-17

**Authors:** Ge Li, Jiang‐Zhi Chen, Shi Chen, Sheng‐Zhe Lin, Wei Pan, Ze‐Wu Meng, Xin‐Ran Cai, Yan‐Ling Chen

**Affiliations:** ^1^ Department of Hepatobiliary Surgery and Fujian Institute of Hepatobiliary Surgery Fujian Medical University Union Hospital Fuzhou China; ^2^ Department of Hepatobiliary Surgery Fujian Provincial Hospital Fuzhou China

**Keywords:** cancer‐specific survival, decision curve analysis, nomogram, overall survival, pancreatic ductal adenocarcinoma

## Abstract

**Background/Aims:**

Pancreatic ductal adenocarcinoma (PDAC) is associated with high mortality, even after surgical resection. The existing predictive models for survival have limitations. This study aimed to develop better nomograms for predicting overall survival (OS) and cancer‐specific survival (CSS) in PDAC patients after surgery.

**Methods:**

A total of 6323 PDAC patients were retrospectively recruited from the Surveillance, Epidemiology, and End Results (SEER) database and randomly allocated into training, validation, and test cohorts. Multivariate Cox regression analysis was conducted to identify significant independent factors for OS and CSS, which were used for construction of nomograms. The performance was evaluated, validated, and compared with that of the 8th edition AJCC staging system.

**Results:**

Ten independent factors were significantly correlated with OS and CSS. The 1‐, 3‐, and 5‐year OS rates were 40%, 20%, and 15%, and 1‐, 3‐, and 5‐year CSS rates were 45%, 24%, and 19%, respectively. The nomograms were calibrated well, with c‐indexes of 0.640 for OS and 0.643 for CSS, respectively. Notably, relative to the 8th edition AJCC staging system, the nomograms were able to stratify each AJCC stage into three prognostic subgroups for more robust risk stratification. Furthermore, the nomograms achieved significant clinical validity, exhibiting wide threshold probabilities and high net benefit. Performance assessment also showed high predictive accuracy and reliability.

**Conclusions:**

The predictive ability and reliability of the established nomograms have been validated, and therefore, these nomograms hold potential as novel approaches to predicting survival and assessing survival risks for PDAC patients after surgery.

## Graphical abstract

In the present study, we constructed and validated nomograms for predicting the 1‐, 3‐, and 5‐year OS and CSS rates after surgical resection of PDAC. The nomograms established in the present study showed good performance for the prediction of OS and CSS, which was superior to that of the existing, conventional AJCC staging system with an improved net benefit. Therefore, these nomograms hold promise as novel prognosis models for improving the prediction of individualized postoperative survival and improving the care of PDAC patients following surgical resection.

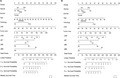

## 1 INTRODUCTION

Pancreatic ductal adenocarcinoma (PDAC) is the most common cancer of the pancreas, accounting for up to 95% of pancreatic cancer cases, and it arises from the malignant transformation of the ductal cells. PDAC is also among the leading causes of cancer‐related death.^1^ The prognosis of PDAC patients is very poor, and the mortality rate remains quite high. It has been estimated that more than 53 000 incident cases develop each year and that the number of deaths due to PDAC can reach approximately 41 000 deaths yearly. As the incidence and the mortality rates of pancreatic cancer have been on the rise recent decades, PDAC is expected to be the second leading cause of cancer‐related death in 2030.^2^ Although a number of therapies have become available for PDAC patients, complete surgical resection is considered the only potentially curative treatment. However, the overall 5‐year survival rate even after surgical resection is reportedly as low as 20%, with only 5%‐7% of patients reaching the median survival time of 25‐30 months.^3^, ^4^, ^5^, ^6^ Clearly, it is essential to improve the clinical outcomes of PDAC patients after surgery by personalizing treatment for each patient.

A prognostic model could facilitate clinical counseling and guide physicians in making treatment and follow‐up plans. Currently, the American Joint Committee on Cancer (AJCC) staging system is widely used as a basis for clinical interventions. However, the AJCC staging system has limitations, as only tumor size and the histological presence are taken into account without consideration of some clinically important prognostic factors, including age, gender, ethnicity, marital status, tumor differentiation, lymph node count (LNC), lymph node ratio (LNR), histology, and grade, differentiation. These limitations may diminish the accuracy of the TNM edition for prognostic evaluation. Thus, a better prognostic model that integrates more clinically important factors is urgently needed.

In this study, we aimed to discover comprehensive factors significantly associated with survival among PDAC patients after surgery and to construct better nomograms for predicting survival, including overall survival (OS) and cancer‐specific survival (CSS), in PDAC patients after surgical resection. Such nomograms should improve the prediction of individualized postoperative survival and thus useful for improving the care of PDAC patients following surgical resection.

## 2 METHODS

### 2.1 Study subjects

In this study, we focused on patients who had been pathologically diagnosed with PDAC after surgical resection. All study subjects were identified from the SEER database (SEER*Stat version 8.3.4) during the period between 2004 and 2015. In the enrollment, the following criteria were used: (a) underwent cancer‐directed surgical resection of primary PDAC; (b) complete information was provided about the T/N/M stage and LNC/LNR; (c) histology codes with 8140, 8141, 8142, 8143, 8144, 8145, 8146, and 8147, according to the International Classification of Disease 3rd edition (ICD‐O3); and (d) data were provided for OS and CSS. The patients who met the following criteria were excluded from this study: (a) PDAC as secondary cancer; (b) absence or incomplete information about survival, follow‐up period, cause of death, or other important clinical characteristics; and (c) PDAC suspected but not pathologically diagnosed. Ultimately, a total of 6323 patients were enrolled. According to machine learning, when the size of the given data is ten thousand and below, the ratio of 7:3 is generally used in the training and test sets, and the ratio of 6:2:2 is usually used in the training, test, and verification sets. Hence, patients in this study were randomly assigned into three cohorts: training cohort (n = 3700), validation cohort (n = 1312), and prospective test cohort (n = 1311) at a ratio of 6:2:2. For group assignment, the data were entered in Excel, and random numbers were generated with the Excel random number generator algorithm. The medical records of the enrolled patients for the following demographical and clinical characteristics were reviewed and analyzed: age, gender, race, marital status, age at diagnosis of PDAC, tumor size, tumor TNM stage, tumor histological findings, cancer‐specific death, and vital status. The LNR was defined and calculated by dividing the number of metastatic nodes by the LNC. The histological features were classified as well/moderately differentiated or poorly differentiated/anaplastic differentiated PDAC.

The requirements of institutional review board (IRB) approval and written informed consent were waived due to the retrospective nature of the study of cases in the publicly available SEER database. All authors had signed authorization and were allowed to access the dataset in the SEER database.

### 2.2 Construction of nomograms

Nomograms were constructed based on the training cohort. The chi‐square test was used for comparisons of categorical variables. To determine the optimal number of knots, linear assumptions of the continuous variables, including age, LNR and LNC, were relaxed using restricted cubic spline functions, in which goodness of fit was maximized using the log‐likelihood, while information loss was minimized with the Akaike information criterion (AIC).^7^ Univariate and multivariate analyses were conducted using the Cox regression model and hazard ratio (HR), respectively. Subsequently, 95% confidence intervals (CIs) were calculated. Based upon the AIC of the Cox proportional hazards model, variables were selected in a backward stepwise manner. Independent variables identified by the multivariate analysis were used for construction of nomograms.

### 2.3 Validation of nomograms and performance assessment

Model performance was assessed using the concordance index (c‐index), in which the values of the C‐index ranged 0.5‐1, with 0.5 considered no discrimination at all and 1.0 representing perfect discrimination. Calibration was made by comparing the means of predicted survival with those of actual survival using observed Kaplan‐Meier estimates. To reduce potential bias, 200‐sample bootstrap validation was performed for internal testifying. The values of c‐indexes were compared using the compare C package.^8^ The precision of the 1‐, 2‐, and 3‐year survival rates predicted by the nomograms was evaluated with time‐dependent receiver‐operating characteristic (ROC) curve analysis using the time ROC^9^ package.

### 2.4 Kaplan‐Meier survival curve and decision curve analyses

The nomograms established in this study were compared with the AJCC8 staging system by risk classification and stratification using Kaplan‐Meier survival curves. For risk stratification, Nomo stages were built by ranking scores of the accumulated nomograms scores by deciles to develop 10 risk groups, and each AJCC8 substage was divided by nomo stages to derive three prognostic strata: low, median, and high risk. Mosaic plots were created to determine the distributions between the AJCC8 stages and the Nomo stages. The ranges of threshold probabilities were finalized by decision curve analysis (DCA)^10^ for the clinical validity of nomograms.^10^

The PASW 18.0 program (SPSS Inc) and the R program (v 3.2.3) using rms^7^ were used for statistical analysis. A two‐tailed *P* value less than .05 indicated statistical significance.

## 3 RESULTS

### 3.1 Baseline demographic and clinical characteristics of the study subjects

A total of 6323 patients with PDAC were enrolled in this study, and the baseline demographic and clinical characteristics of the study subjects are summarized in Table 1. All characteristics were comparable among the patients in the different cohorts. The median follow‐up period was 15 months (range, 1‐143 months). Among 2716 (73.4%) patients who died during the period of follow‐up, 2389 cancer‐specific deaths and 327 non–cancer‐specific deaths were identified. The 1‐, 3‐, and 5‐year OS rates were 40%, 20%, and 15%, respectively, and the 1‐, 3‐, and 5‐year CSS rates were 45%, 24%, and 19%, respectively.

**TABLE 1:** Baseline demographic and clinical characteristics of the patients

Variables	Training cohort (n = 3700)	Validation cohort (n = 1312)	Test cohort (n = 1311)	*P* value
Gender, n (%)				
Female	1824 (49.3)	649 (49.5)	637 (48.6)	.884
Male	1876 (50.7)	663 (50.5)	674 (51.4)	
Age (y), median (range)	66 (18‐99)	66 (18‐99)	66 (18‐99)	.76
Race, n (%)				
White	3047 (82.3)	1087 (82.9)	1088 (82.9)	.135
Black	389 (10.5)	157 (12.1)	129 (9.8)	
Asian (Chinese, Korean, and Japanese)	117 (3.1)	30 (2.2)	37 (2.8)	
Other	147 (3.9)	38 (2.8)	57 (4.3)	
Marital status at diagnosis, n (%)				
Married (including separated)	2320 (62.7)	828 (63.1)	815 (62.1)	.164
Divorced	414 (11.1)	142 (10.8)	131 (9.9)	
Single (never married)	425 (11.4)	161 (12.2)	135 (10.2)	
Widowed	438 (11.8)	145 (11)	180 (13.7)	
Unknown	103 (2.7)	36 (2.7)	50 (3.8)	
Tumor size, n (%)				
≤2 cm	576 (15.6)	218 (16.6)	235 (17.9)	.443
>2 and ≤4 cm	2221 (60)	791 (60.3)	751 (57.3)	
>4 cm	903 (24.4)	303 (23.1)	325 (24.8)	
Extent of surgery, n (%)				
PP	465 (12.6)	177 (13.5)	168 (12.8)	.909
PD	2785 (75.3)	984 (75)	987 (75.3)	
TP	450 (12.1)	151 (11.5)	156 (11.9)	
Histology, n (%)				
Well/moderately differentiated	2050 (55.4)	710 (54.1)	730 (55.7)	.368
Poorly differentiated/anaplastic	1313 (35.5)	500 (38.1)	469 (35.8)	
Unknown	337 (9.1)	102 (7.8)	112 (8.5)	
pT stage, n (%)				
pT1	564 (15.2)	214 (16.3)	228 (17.4)	.437
pT2	2119 (57.3)	746 (56.9)	712 (54.3)	
pT3	814 (22)	274 (20.9)	292 (22.3)	
pT4	203 (5.5)	78 (5.9)	79 (6)	
pN stage, n (%)				
N0	1325 (35.8)	471 (35.9)	450 (34.3)	.501
N1	1484 (40.1)	518 (34.3)	559 (42.6)	
N2	891 (24.1)	323 (24.6)	302 (23)	
Lymph node count, mean (SD)	15.6 (9.8)	15.4 (9.9)	15.3 (9.9)	.623
Lymph node ratio, mean (IQR)	0.17 (0‐0.25)	0.17 (0‐0.26)	0.16 (0‐0.25)	.769
Metastasis, n (%)				
M0	3497 (94.5)	1268 (96.6)	1238 (94.4)	.007
M1	203 (5.5)	44 (3.4)	73 (5.6)	
1‐y cumulative survival	0.4 (0.45)	0.42 (0.63)	0.41 (0.46)	
3‐y cumulative survival	0.2 (0.24)	0.23 (0.84)	0.21 (0.25)	
5‐y cumulative survival	0.15 (0.19)	0.15 (0.81)	0.15 (0.2)	

Abbreviations: PD, pancreaticoduodenectomy; PP, partial pancreatectomy; TP, total pancreatectomy.

### 3.2 Univariate and multivariate analyses of factors associated with OS and CSS

Univariate and multivariate Cox regression analyses were performed to identify factors significantly correlated with OS and CSS, and the results are presented in Tables 2 and 3. On the univariate analysis, older age (*P* < .001), marital status (*P* < .05), larger tumor size (*P* < .001), more advanced TNM 8th T and N stages (both *P* < .001), poorly differentiated status (*P* < .001), metastatic disease (*P* < .001), PLNC (*P* < .001), LNC (*P* < .001), and LNR (*P* < .001) were significantly associated with OS and CSS (Table 2). The variables that showed significant association with OS and CSS on the univariate analyses were used for subsequent multivariate analysis to determine the factors independently related to OS and CSS (Table 3).

**TABLE 2:** Univariate Cox regression analysis of the patients in the training group

Variables	OS			CSS		
	HR	95% CI	*P*	HR	95% CI	*P*
Marital status at diagnosis	0.884	0.820‐0.954	.002	0.882	0.813‐0.956	.002
Single	ref			ref		
Married	0.937	0.828‐1.062	.308	0.956	0.838‐1.090	.503
Divorced	1.08	0.955‐1.222	.085	1.1	0.966‐1.253	.106
Widowed	1.191	1.062‐1.336	.003	1.137	1.004‐1.288	.041
Unknown	0.833	0.646‐1.073	.398	0.837	0.640‐1.096	.372
Race						
Black	ref			ref		
White	1.03	0.915‐1.169	.59	0.997	0.873‐1.14	.968
Yellow	0.891	0.715‐1.111	.233	0.942	0.749‐1.185	.665
Other	0.818	0.667‐1.003	.047	0.871	0.705‐1.076	.272
Gender						
Female	ref			ref		
Male	0.901	0.835‐0.971	.006	0.905	0.835‐0.981	.015
Age	1.247	1.181‐1.317	<.0001	1.158	1.093‐1.227	<.0001
Histology						
Well/moderately differentiated	ref			ref		
Poorly differentiated/anaplastic	1.363	1.258‐1.475	<.0001	1.416	1.301‐1.541	<.0001
Unknown	0.884	0.763‐1.025	.1016	0.912	0.780‐1.067	.2493
LNC	0.891	0.848‐0.935	<.0001	0.914	0.868‐0.962	.001
Tumor size	1.168	1.132‐1.205	<.0001	1.161	1.122‐1.200	<.0001
LNR	1.377	1.324‐1.433	<.0001	1.411	1.353‐1.471	<.0001
TNM 8th stage						
IA	ref			ref		
IB	0.474	0.398‐0.566	0	0.442	0.364‐0.536	.001
IIA	0.672	0.602‐0.750	<.0001	0.635	0.564‐0.715	<.0001
IIB	0.862	0.729‐1.019	<.0001	0.771	0.639‐0.929	<.0001
III	1.211	1.102‐1.332	<.0001	1.248	1.130‐1.379	<.0001
IV	1.78	1.519‐2.086	<.0001	1.79	1.514‐2.118	<.0001
pT stage						
T1	ref			ref		
T2	0.721	0.643‐0.809	<.0001	0.716	0.634‐0.809	<.0001
T3	1.289	1.176‐1.413	<.0001	1.257	1.139‐1.388	<.0001
T4	1.487	1.264‐1.750	<.0001	1.475	1.240‐1.755	<.0001
pN stage						
N0	ref			ref		
N1	0.66	0.604‐0.721	<.0001	0.616	0.560‐0.678	<.0001
N2	1.232	1.121‐1.353	<.0001	1.28	1.160‐1.413	<.0001
pM stage						
M0	ref			ref		
M1	1.964	1.688‐2.285	<.0001	1.996	1.700‐2.344	<.0001

Abbreviations: 95% CI, 95% confident interval; CSS, cancer‐specific survival; HR, hazard ratio; LNC, lymph node count; LNR, lymph node ratio; OS, overall survival; ref, reference category.

**TABLE 3:** Multivariate Cox regression analysis of the patients in the training group

Covariates	OS			CSS		
	HR	95% CI	*P*	HR	95% CI	*P*
Marital status at diagnosis (ref, Single)	ref			ref		
Married	1.24	1.170‐1.315	.92	1.152	1.083‐1.225	.902
Divorced	0.875	0.825‐0.929	.014	0.884	0.831‐0.941	.013
Widowed	1.051	0.999‐1.106	.04	1.047	0.991‐1.106	.062
Unknown	1.184	1.109‐1.264	.601	1.188	1.109‐1.273	.619
Gender (female vs male)	0.994	0.876‐1.127	.001	0.992	0.868‐1.133	.0056
Age	1.218	1.075‐1.379	<.0001	1.233	1.081‐1.407	<.0001
Histology (ref, Well/moderately differentiated)						
Poorly differentiated/anaplastic	1.181	1.042‐1.337	<.0001	1.173	1.025‐1.342	<.0001
Unknown	0.923	0.716‐1.191	.1503	0.921	0.703‐1.207	.3891
LNC	0.896	0.771‐1.041	<.0001	0.933	0.796‐1.093	<.0001
Tumor size	1.307	1.207‐1.416	.054	1.358	1.248‐1.479	.0996
LNR	0.896	0.771‐1.041	<.0001	0.933	0.796‐1.093	<.0001
pT stage (ref, pT1)						
T2	0.796	0.702‐0.903	.0004	0.802	0.701‐0.918	.001
T3	1.121	0.992‐1.267	.0003	1.089	0.955‐1.242	.002
T4	1.354	1.132‐1.619	<.0001	1.333	1.103‐1.612	<.0001
pN stage (ref, pN0)						
N1	0.748	0.673‐0.832	<.0001	0.703	0.639‐0.802	<.0001
N2	1.086	0.967‐1.221	<.0001	1.1285	0.998‐1.276	<.0001
Metastasis (M1 vs M0)	1.762	1.506‐2.061	<.0001	1.792	1.518‐2.117	<.0001

Abbreviations: 95% CI, 95% confident interval; CSS, cancer‐specific survival; HR, hazard ratio; LNC, lymph node count; LNR, lymph node ratio; OS, overall survival; ref, reference category.

### 3.3 Construction and calibration of nomograms for OS and CSS

**FIGURE 1:**
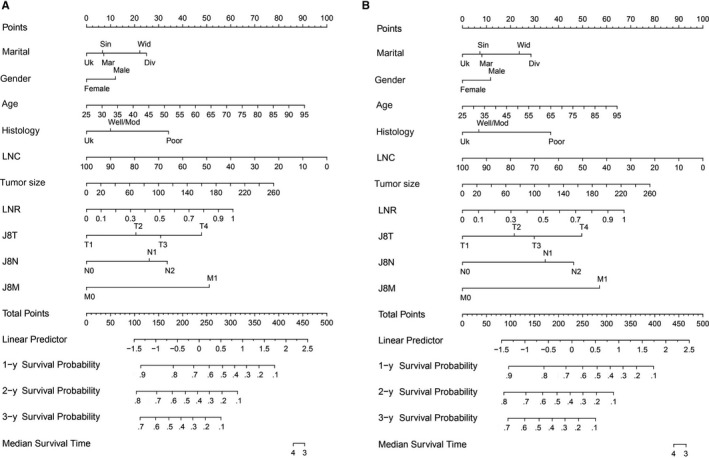
Nomograms for the prediction of survival in pancreatic ductal adenocarcinoma patients after surgery. (A) 1‐, 3‐, and 5‐y cancer‐specific survival; (B) 1‐, 3‐, and 5‐y overall survival. Covariates in each nomogram were assessed for the patient and given a point. A higher total number of points represented a higher possibility of unfavorable clinical outcomes and a lower expected survival

The significant independent factors identified in the training cohort were used in the construction of nomograms for predicting OS and CSS (Figure 1). The probability of individual survival was calculated by adding up all scores for each selected variable. The details of labels and points in the nomogram are presented in Tables 4 and 5.

**TABLE 4:** Points for categorical variables in nomograms

Variables	OS	Variables	CSS
*Marital status*	*Points*	*Marital status*	*Points*
Single	7	Single	7
Married	7	Married	8
Divorced	25	Divorced	28
Widowed	22	Widowed	24
Unknown	0	Unknown	0
*Gender*	*Points*	*Gender*	*Points*
Female	0	Female	0
Male	12	Male	12
*Histology*	*Points*	*Histology*	*Points*
Well/moderately differentiated	10	Well/moderately differentiated	7
Poorly differentiated/anaplastic	34	Poorly differentiated/anaplastic	37
Unknown	0	Unknown	0
*J8T*	*Points*	*J8T*	*Points*
T1	0	T1	0
T2	21	T2	22
T3	31	T3	30
T4	48	T4	50
*J8N*	*Points*	*J8N*	*Points*
N0	0	N0	0
N1	26	N1	34
N2	34	N2	46
*J8M*	*Points*	*J8M*	*Points*
M0	0	M0	0
M1	51	M1	57

Abbreviations: CSS, cancer‐specific survival; OS, overall survival.

**TABLE 5:** Points for continuous variables in the nomograms

Age (y)	Points		LNC	Points		Tumor size	Points		LNR	Points	
	OS	CSS		OS	CSS		OS	CSS		OS	CSS
25	0	0	0	100	100	0	0	0	0	0	0
30	6	5	10	90	90	20	6	6	0.1	6	7
35	13	9	20	80	80	40	12	12	0.2	12	13
40	19	14	30	70	70	60	18	18	0.3	18	20
45	26	18	40	60	60	80	24	24	0.4	24	27
50	32	23	50	50	50	100	30	30	0.5	30	34
55	39	28	60	40	40	120	36	36	0.6	37	40
60	45	32	70	30	30	140	42	42	0.7	43	47
65	52	37	80	20	20	160	48	48	0.8	49	54
70	58	41	90	10	10	180	54	54	0.9	55	60
75	65	46	100	0	0	200	60	60	1	61	67
80	71	51				220	66	66			
85	78	55				240	72	72			
90	84	60				260	78	78			
95	91	64									

Abbreviations: CSS, cancer‐specific survival; LNC, lymph node count; LNR, lymph node ratio; OS, overall survival.

**FIGURE 2:**
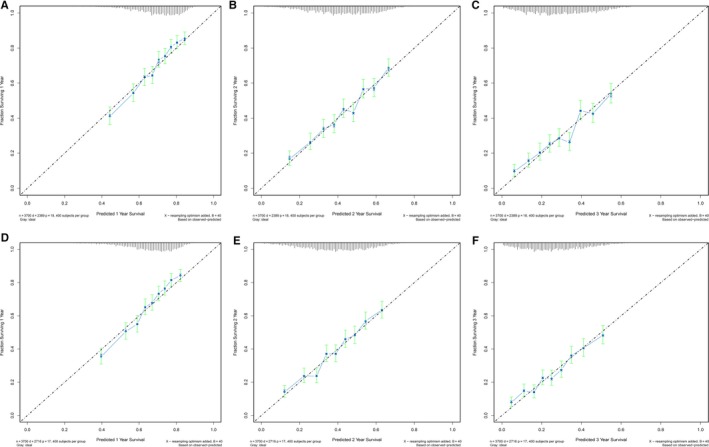
Bootstrap calibrations of the nomograms. Bootstrap calibrations of the nomograms for predicting (A) 1‐y CSS; (B) 3‐y CSS; (C) 5‐y CSS; (D) 1‐y OS; (E) 3‐y OS; and (F) 5‐y OS, which were well correlated with the actual survival probabilities. CSS, cancer‐specific survival; OS, overall survival

The bootstrap‐corrected c‐indexes in the training cohort provided good validation (CSS, 0.6425; OS, 0.6404). Calibration plots were generated for the probabilities of 1‐, 3‐, and 5‐year OS and CSS and showed optimal agreement between the survival predicted by the nomogram and the corresponding Kaplan‐Meier estimates in the training cohorts (Figure 2), indicating that the two cohorts were calibrated well and the established nomograms were reliable for predicting survival in PDAC patients after surgical resection.

### 3.4 External validation of nomograms

**FIGURE 3:**
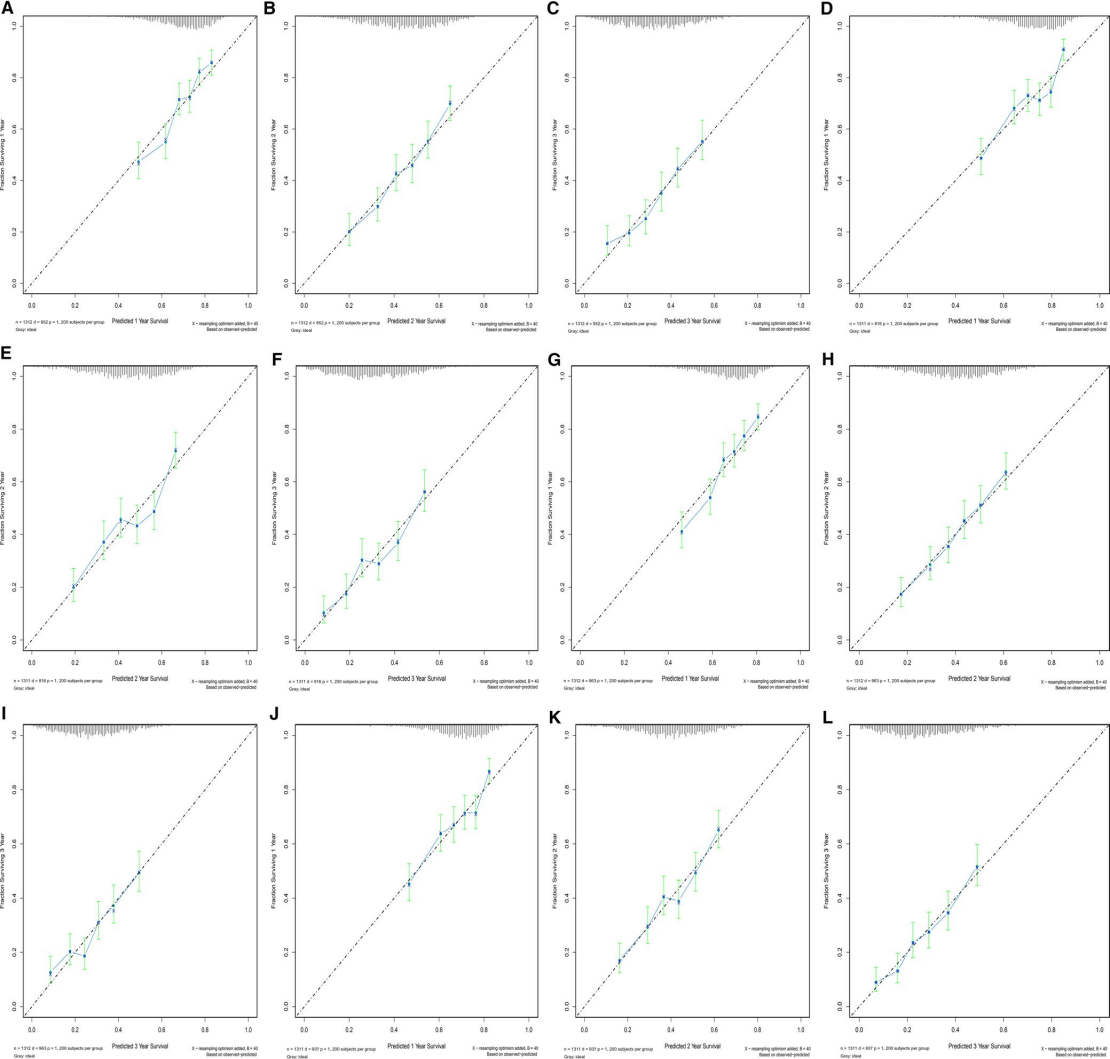
Bootstrap calibration of nomograms in the validation cohorts. The nomograms were externally validated in the validation cohorts by predicting (A) 1‐y CSS; (B) 3‐y CSS; and (C) 5‐y CSS, and in the test cohorts by predicting (D) 1‐y CSS; (E) 3‐y CSS; and (F) 5‐y CSS. Similarly, the nomograms were externally validated in the validation cohorts by predicting (G) 1‐y OS; (H) 3‐y OS; and (I) 5‐y OS, and in the test cohorts by predicting (J) 1‐y OS; (K) 3‐y OS; and (L) 5‐y OS. All results showed good validation. CSS, cancer‐specific survival; OS, overall survival

The c‐indexes of the nomograms for predicting CSS and OS were 0.632 (95% CI 0.611‐0.652) and 0.631 (95% CI 0.611‐0.650) in the validation cohort, respectively. In the test cohort, the c‐indexes were 0.625 (95% CI 0.603‐0.646) and 0.623 (95% CI 0.603‐0.644), respectively (Table 6). The external calibration plots also showed good validation (Figure 3).

**TABLE 6:** Comparison of nomogram with the AJCC staging system

	Nomogram score	8th AJCC stage	*P*
Training cohort			
OS			
AIC	39 676.98	39 965.12	
Log‐likelihood	−19 837.49	−19 977.56	All <.0001
c‐index	0.640 (0.629‐0.652)	0.585 (0.573‐0.597)	All <.0001
CSS			
AIC	34 980.72	35 197.17	
Log‐likelihood	−17 489.36	−17 593.58	All <.0001
c‐index	0.643 (0.630‐0.655)	0.594 (0.582‐0.607)	All <.0001
Validation cohort			
OS			
AIC	12 068.95	12 118.45	
Log‐likelihood	−6033.474	−6058.226	All <.0001
c‐index	0.631 (0.611‐0.650)	0.595 (0.576‐0.615)	All <.0001
CSS			
AIC	10 734.43	10 751.25	
Log‐likelihood	−5366.215	−5374.626	All <.0001
c‐index	0.632 (0.611‐0.652)	0.604 (0.583‐0.624)	All <.0001
Test cohort			
OS			
AIC	11 704.87	11 804.16	
Log‐likelihood	−5851.434	−5901.081	All <.0001
c‐index	0.623 (0.603‐0.644)	0.582 (0.561‐0.602)	All <.0001
CSS			
AIC	10 241.45	10 319.96	
Log‐likelihood	−5119.723	−5158.978	All <.0001
c‐index	0.625 (0.603‐0.646)	0.589 (0.567‐0.611)	All <.0001

Abbreviations: AJCC, American Joint Committee on Cancer; CSS, cancer‐specific survival; OS, overall survival.

### 3.5 Comparative analysis of the predictive performance between the nomogram and 8th edition TNM staging systems

The areas under the ROC curves (AUCs) for predicting CSS ranged from 66.77% to 70.03% in the three cohorts from 1 to 3 years. As shown in Table 7, the AUCs for the prediction of OS varied from 66.62% to 69.74% during the same years. Thus, the nomograms exhibited considerable discriminatory capacity.

**TABLE 7:** Time‐dependent ROC curve analysis

Study cohort	OS AUC (%)						CSS AUC (%)					
	1 y	95% CI	2 y	95% CI	3 y	95% CI	1 y	95% CI	2 y	95% CI	3 y	95% CI
Training cohort	69.61	67.84‐71.61	68.47	66.86‐70.66	67.98	66.19‐70.71	70.03	68.12‐72.07	68.26	66.59‐70.50	67.83	65.99‐70.59
Validation cohort	68.85	65.68‐72.14	68.13	65.10‐71.47	66.63	63.31‐70.93	69.08	65.80‐72.47	68.32	65.18‐71.70	66.77	63.34‐71.10
Test cohort	66.62	63.01‐69.73	67.33	63.98‐70.48	69.74	66.40‐73.73	66.94	63.15‐70.14	67.31	63.74‐70.46	69.62	66.12‐73.63

Abbreviations: 95% CI, 95% confident interval; AUC, areas under curves; CSS, cancer‐specific survival; OS, overall survival; ROC, receiver‐operating characteristic.

### 3.6 Comparative analysis of the predictive performance between nomograms and AJCC stages

**FIGURE 4:**
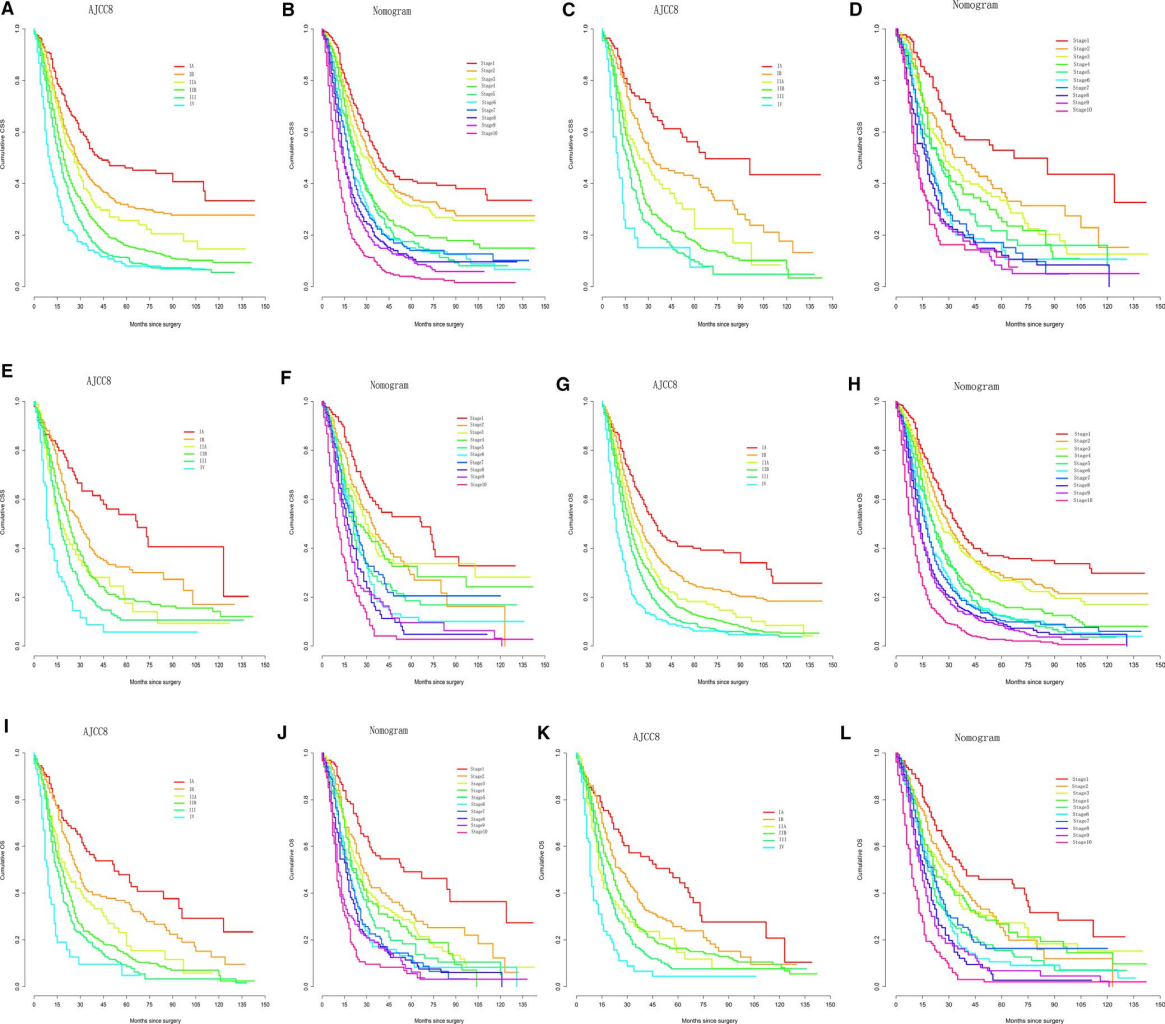
Kaplan‐Meier curve analysis of risk classification. Risk classification of cancer‐specific survival (CSS) in the (A, B) training cohort, (C, D) validation cohort, and (E, F) test cohort. Risk classification of overall survival (OS) in the (G, H) training cohort, (I, J) validation cohort, and (K, L) test cohort. All log‐rank *P* values for trends were <.0001

**FIGURE 5:**
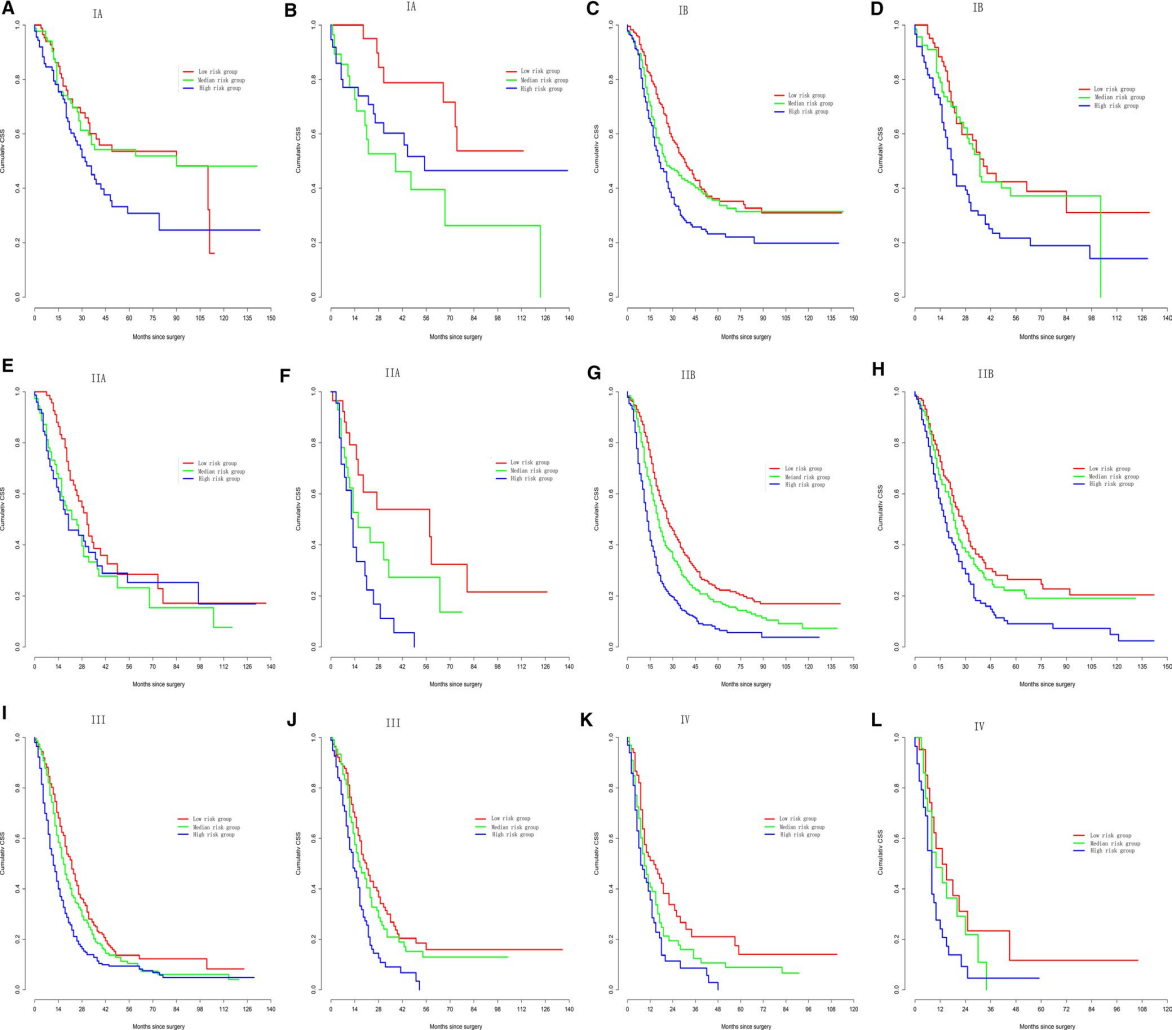
Kaplan‐Meier curve analysis of risk stratification. Risk stratification of cancer‐specific survival (CSS) for each AJCC8 substages in the (A, C, E, G, I, K) training cohort and the (B, D, F, H, J, L) test cohort. All log‐rank *P* values for trends were <.005, except for (E) and (G) (*P* = .1)

**FIGURE 6:**
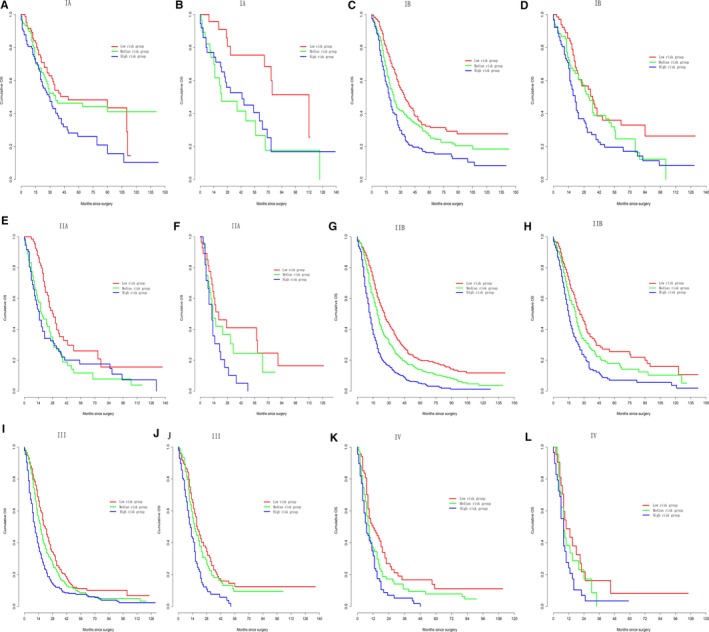
Kaplan‐Meier curve analysis of risk stratification. Risk stratification of overall survival (OS) for each AJCC8 substages in the (A, C, E, G, I, K) training cohort and the (B, D, F, H, J, L) test cohort. All log‐rank *P* values for trends were <.005, except for (I) (*P* = .2)

**FIGURE 7:**
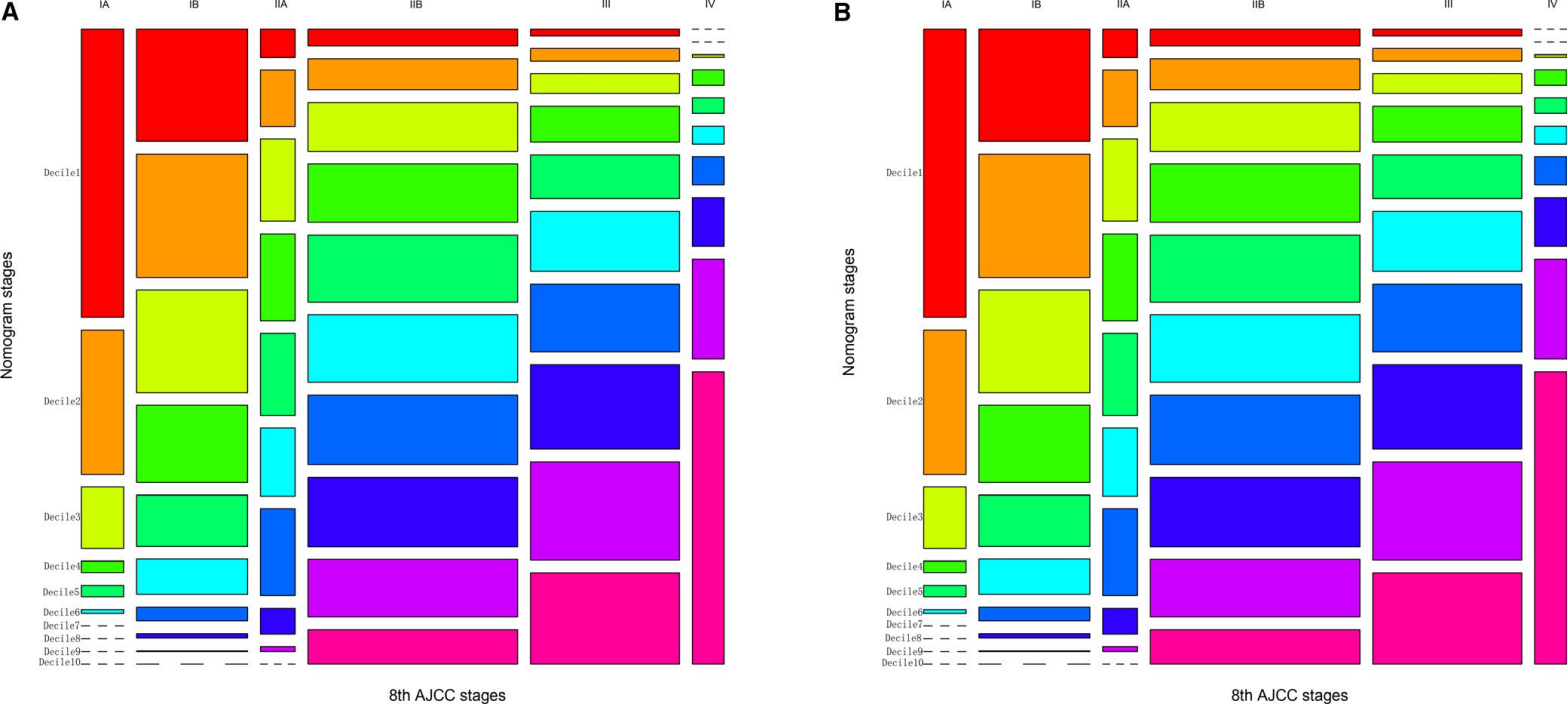
Mosaic plots using the training cohort. (A) Mosaic plots for cancer‐specific survival and (B) mosaic plots for overall survival in which each of the 10 deciles was represented by 1 of 10 consecutive rainbow colors. The area of the individual mosaics represented the relative frequency associated with the column cell. The short segmented lines indicated a frequency of zero. AJCC, American Joint Committee on Cancer

First, the nomograms showed the greatest log‐likelihoods and c‐indexes, together with the smallest values of AIC for CSS and OS prediction in all three cohorts in comparison to the AJCC 8th stages (Table 6). These results suggest that the nomograms were better and more robust for survival prediction than the AJCC stages. Second, according to the points in the nomogram, the nomograms stratified patients into 10 Nomo stages, which showed a better ability to discriminate OS and CSS as compared with the AJCC8 stages illustrated by the Kaplan‐Meier curves, especially within 5 years after surgery (Figure 4). The 3‐year cumulative survival (Table 8) and HRs (Table 9) of the Nomo stages also confirmed the classification ability of the nomograms. Further analysis (Figures 5 and 6) showed that the nomograms were also capable of stratifying each AJCC8th stage into the following three significant groups for prognosis: low‐, medium‐, and high‐risk groups, demonstrating a good ability for risk stratification. Finally, the dramatic survival heterogeneity between the 8th edition AJCC substages and the Nomo stages was demonstrated intuitively by mosaic plots (Figure 7), indicating the underlying frequencies of staging limitations in the traditional AJCC staging system.

**TABLE 8:** Cumulative survival for Nomo stages in derivation and external validation cohorts

Nomo stage	Training cohort (cumulative survival, 36 mo, %)				Validation cohort (cumulative survival, 36 mo, %)				Test cohort (cumulative survival, 36 mo, %)			
	OS	95% CI	CSS	95% CI	OS	95% CI	CSS	95% CI	OS	95%CI	CSS	95% CI
nomo1	48.00	42.20‐53.70	53.20	47.10‐58.90	50.50	38.54‐61.20	53.20	47.10%‐58.90	54.60	42.51‐65.20	59.10	47.0‐70.3
nomo2	41.50	35.90‐47.10	44.90	39.10‐50.50	47.57	37.66‐56.80	44.90	39.10%‐50.50	46.80	36.53‐56.40	49.30	37.8‐58.2
nomo3	41.90	36.30‐47.40	46.20	40.30‐51.90	38.50	29.41‐47.50	46.20	40.30%‐51.90	32.40	23.34‐41.70	36.80	30.4‐51.7
nomo4	26.04	21.15‐31.20	29.60	24.22‐35.10	38.96	30.21‐47.60	29.60	24.22%‐35.10	39.50	30.20‐48.60	42.00	30.8‐50.2
nomo5	24.97	20.23‐29.99	28.92	23.72‐34.30	28.42	20.36‐37.00	28.92	23.72%‐34.30	24.68	17.29‐32.78	28.73	18.9‐34.9
nomo6	20.92	16.39‐25.83	26.52	21.20‐32.10	15.70	9.63‐23.10	26.52	21.20%‐32.10	25.31	17.76‐33.50	28.30	21.9‐39.9
nomo7	19.28	14.99‐24.00	22.10	17.28‐27.20	18.41	11.84‐26.10	22.10	17.28%‐27.20	21.50	14.17‐29.80	24.00	18.9‐36.1
nomo8	14.91	11.05‐19.31	17.84	13.34‐22.90	21.76	14.84‐29.60	17.84	13.34%‐22.90	15.19	9.32‐22.40	19.04	9.1‐22.7
nomo9	13.52	10.00‐17.57	15.55	11.57‐20.06	18.93	12.43‐26.48	15.55	11.57%‐20.06	13.93	7.85‐21.73	16.45	8.6‐24.7
nomo10	8.27	5.60‐11.57	10.57	7.30‐14.53	8.87	4.45‐15.17	10.57	7.30%‐14.53	5.55	2.32‐10.85	7.68	4.4‐15.9
*P*log‐rank for trend	<.0001	<.0001			<.0001	<.0001			<.0001	<.0001		

Abbreviations: 95% CI, 95% confident interval; CSS, cancer‐specific survival; OS, overall survival.

**TABLE 9:** Relative hazard for Nomo stages in derivation and external validation cohorts

Nomo stages	Cut‐point	Training cohort			Validation cohort			Test cohort		
		HR	95% CI	*P*	HR	95% CI	*P*	HR	95% CI	*P*
OS										
nomo1	<182.02	ref			ref			ref		
nomo2	<197.61	1.26	1.04‐1.53	.018	1.64	1.16‐2.33	.0056	1.47	1.05‐2.07	.025
nomo3	<210.90	1.37	1.13‐1.67	.0013	1.95	1.40‐2.72	<.0001	1.56	1.11‐2.19	.0102
nomo4	<221.85	1.82	1.51‐2.19	<.0001	2.14	1.53‐2.99	<.0001	1.623	1.16‐2.29	.0046
nomo5	<233.15	2.05	1.71‐2.47	<.0001	2.48	1.78‐3.47	<.0001	2.24	1.63‐3.09	.0001
nomo6	<243.66	2.30	1.91‐2.76	<.0001	2.97	2.13‐4.13	<.0001	2.35	1.70‐3.25	.0001
nomo7	<255.43	2.40	2.00‐2.89	<.0001	2.99	2.15‐4.16	<.0001	2.06	1.48‐2.86	.0001
nomo8	<268.63	2.87	2.39‐3.45	<.0001	3.34	2.41‐4.64	<.0001	3.03	2.19‐4.19	<.0001
nomo9	<288.77	3.14	2.62‐3.77	<.0001	4.04	2.90‐5.63	<.0001	3.18	2.30‐4.40	<.0001
nomo10	288.77+	4.93	4.12‐5.91	<.0001	5.36	3.84‐7.48	<.0001	5.23	3.79‐7.22	<.0001
CSS										
nomo1	<170.82	ref			ref			ref		
nomo2	<187.69	1.24	1.01‐1.52	.043	1.56	1.07‐2.27	.02	1.54	1.06‐2.22	.0227
nomo3	<202.07	1.35	1.10‐1.66	.0042	1.86	1.31‐2.66	.0006	1.54	1.06‐2.24	.0243
nomo4	<213.92	1.74	1.42‐2.13	<.0001	2.10	1.47‐3.00	<.0001	1.69	1.16‐2.44	.0057
nomo5	<226.15	2.02	1.66‐2.45	<.0001	2.40	1.68‐3.43	<.0001	2.33	1.64‐3.30	<.0001
nomo6	<237.53	2.28	1.87‐2.78	<.0001	2.99	2.11‐4.24	<.0001	2.56	1.80‐3.63	<.0001
nomo7	<250.26	2.32	1.91‐2.83	<.0001	3.00	2.12‐4.26	<.0001	2.22	1.56‐3.17	<.0001
nomo8	<264.54	2.82	2.32‐3.43	<.0001	3.45	2.44‐4.88	<.0001	3.18	2.24‐4.52	<.0001
nomo9	<286.33	3.15	2.60‐3.82	<.0001	4.24	3.00‐6.01	<.0001	3.41	2.40‐4.84	<.0001
nomo10	286.33+	4.94	4.08‐5.98	<.0001	4.82	3.37‐6.90	<.0001	5.32	3.74‐7.56	<.0001

### 3.7 Decision curve analysis

**FIGURE 8:**
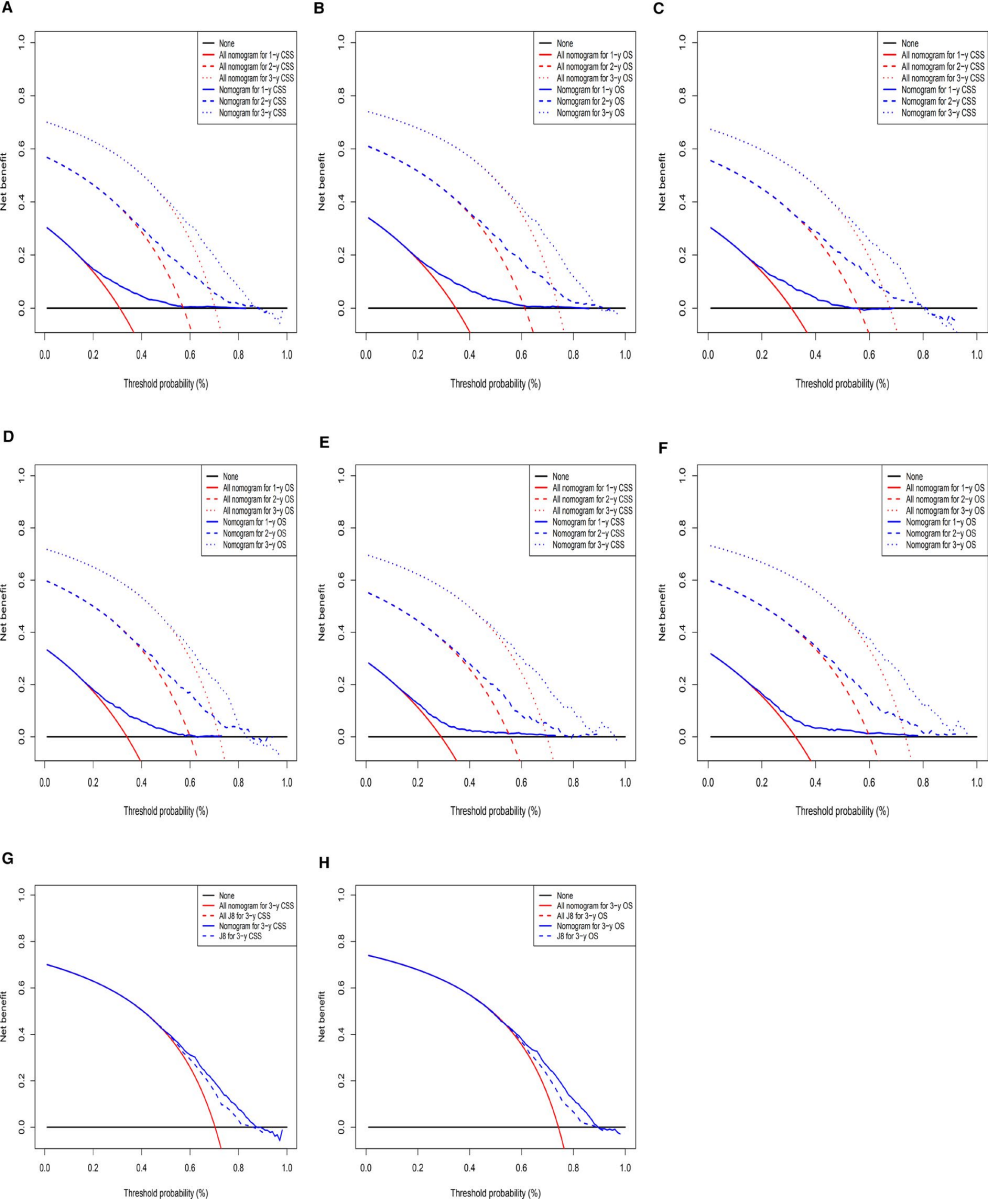
Decision curve analysis and comparison of the nomograms with the AJCC stages. Decision curve analysis of the nomograms for predicting (A) 1‐, 2‐, and 3‐y CSS in the training cohort; (B) 1‐, 2‐, and 3‐y OS in the training cohort; (C) 1‐, 2‐, and 3‐y CSS in the validation cohort; (D) 1‐, 2‐, and 3‐y OS in the validation cohort; (E) 1‐, 2‐, and 3‐y CSS in the test cohort; and (F) 1‐, 2‐, and 3‐y OS in the test cohort; comparison of the nomograms with the 8th version of the AJCC stages for predicting (G) 3‐y CSS in the training cohort; (H) 3‐y OS in the training cohort. Black horizontal lines represented the assumption that events occurred in no patient within a particular timespan. Red lines represented the assumption that events occurred in all patients within the same time span. Blue lines indicated the net benefit of model prediction. The net benefit per patient of each predictive model within a particular time span was a function of the cohort size with threshold probability, and was computed by addition of the benefit (true positive) and subtraction of the harm (false positive). AJCC, American Joint Committee on Cancer; CSS, cancer‐specific survival; OS, overall survival

Decision curve analysis was applied to verify the clinical validity of the nomograms and demonstrated wide ranges of threshold probabilities in all cohorts, showing good clinical applicability of the nomograms for predicting survival of PDAC patients. Additional comparisons between the nomograms and the TNM stages indicated that the net benefit was consistently enhanced in the nomograms, suggesting that the nomograms were superior to the conventional AJCC staging system for the prediction of both OS and CSS among PDAC patients after surgical resection (Figure 8).

## 4 DISCUSSION

Accurate prediction of prognosis is critical for better management of PDAC patients following surgery, and to date, this has mainly relied on the AJCC staging system. However, the predictive accuracy of the existing systems for PDAC, including the updated editions, is unsatisfactory. In fact, even among cases with the same TNM stage, post‐surgical survival for PDAC patients remains heterogeneous.^4^, ^11^, ^12^ This study, on the basis of a large sample size (6323 cases) spanning a long period (12 years), produced the following major novel findings. (a) Ten independent factors were identified to be significantly correlated with OS and CSS in PDAC patients after surgery. (b) Multivariate logistic regression‐based nomograms for predicting OS and CSS were established with c‐indices of 0.640 for OS and 0.643 for CSS. (c) The nomograms were calibrated well in training and validation cohorts. (d) The performance of the nomograms for OS and CSS was better than that of the 8th edition AJCC staging system as more clinical net benefits were obtained. (e) Our results support that the nomograms established in this study had better performance for the prediction of survival and thus hold potential for clinical application in the predication of survival among PDAC patients after surgical resection.

Prognostic nomograms have been widely recognized and well accepted as simple models for prediction of prognosis in which complicated statistical models are represented by elegant graphics.^13^, ^14^, ^15^ In addition, prognostic nomograms have been shown to be more comprehensive and accurate with easily measured clinical characteristics and with feasibility of application in clinical practice in comparison with other models. In this study, we integrated more clinically important prognostic factors into the generation of the nomograms for prediction of survival in PDAC patients who had undergone surgical resection than had been used in a number of previous studies.^11^, ^16^, ^17^, ^18^, ^19^, ^20^, ^21^ The tumor characteristics were thought to be the most important features that can affect survival after pancreatic resection. Of these characteristics, tumor size was the most important factor associated with OS, especially for tumors <3 cm,^22^ for which this category was adopted by the 8th TNM staging system. In addition to tumor size, lymph node status is also recognized as a key prognostic factor in relation with disease‐free survival and OS, although various cutoffs have been used in different studies.^23^, ^24^ According to the latest TNM staging system, the N stages were stratified into N0, N1, and N2. In addition, tumor differentiation^16^ and neural invasion^20^ have been found to be independent factors significantly associated with poor clinical outcomes following surgical removal of PDAC. In this study, multivariate logical regression analyses were performed and identified older age, larger tumor size, poorer differentiation, more advanced T and N stages, metastasis, harvested lymph node numbers, and lymph node ratio as independent factors significantly associated with lower OS rates, and thus poorer prognosis, after surgical resection of PDAC. These findings are in agreement with those of a number of previous studies.^11^, ^16^, ^17^, ^18^, ^19^, ^20^, ^21^ Considering that the surgical procedures performed could be affected by many factors, such as tumor size, location of tumor, condition of patients, and the experience of doctors, we did not include operation as a prognostic factor in this study.

It has been noted that the correlations between some factors and prognosis of patients after surgical removal of PDAC are still matters of debate. For example, the effect of lymph node metastasis on the survival of PDAC patients after surgery has been controversial.^25^ In a previous study,^26^ the LNR as a prognostic reference factor was taken into account, whereas the N stage of version 8 TNM was not considered as an independent risk factor for both OS and CSS. In this study, multivariate analysis revealed that the LNC, LNR, and the TNM8th N stage were strongly correlated with the survival of patients following surgical resection of PDAC, including OS and CSS. The N staging is currently based on the number of lymph node metastases alone. A previous study^27^ demonstrated that patients with two or more positive lymph nodes have a very low 5‐year OS rate of 5% and that those individuals with one or less positive lymph nodes have a 5‐year OS up to 44%. Additionally, in the same study, the TNM 8th N stage showed that more meticulous stratification of the lymph nodes status was associated with more accurate prognosis prediction. The previous findings suggested that the number of the positive lymph nodes has a significant impact on the prognosis, but it was independently associated with OS and CSS in our study. There is a possibility that the number of positive lymph node metastases may be biased by the LNC. Compared with the number of lymph nodes, the LNC and LNR were more closely related to prognosis. Harvesting of 13‐16 lymph nodes was highly recommended for accurate staging and prognosis in a previous study,^28^ whereas another previous study^29^ considered the number to be 20. The LNR incorporates information concerning positive LNs and an estimate of adequate LNs obtained. An elevated LNR may reflect the progression or tendency of metastasis, and for this reason, was found to correlate with poorer OS and CSS in this study.

It may also merit attention in our study that marital status and gender were integrated into the construction of the prognostic nomograms for PDAC for the first time. According to the World Health Organization (WHO), the incidence of pancreatic cancer is higher in men than in women, and this gender difference appears to be even greater in developed countries. It is possible that male patients could be more vulnerable to environmental or genetic factors, and that marriage could make a prognostic difference.^20^

Recently, several nomograms to predict the prognosis of PDAC patients have been reported,^26^, ^30^, ^31^, ^32^ and in comparison, the nomograms established in this study show a number of strengths. First, we developed nomograms for the prediction of survival based on the analysis of 6323 patients with PDAC, a larger sample size than used in previous studies. Second, internal and external validations were performed in our study, suggesting agreement between the predicted and actual survival. Third, we also selected covariates based upon the AIC and likelihood rather than statistical significance (*P* value) to balance model complexity and performance. Fourth, to avoid missing information caused by categorization, we adopted restricted cubic splines for those continuous variables in this study.^7^ Fifth, this is the first attempt to build nomograms that can stratify each AJCC stage into three prognostic subgroups to achieve better and more robust risk stratification for the PDAC patients. The risk classification and stratification are important, as they may support a better understanding of the degree of survival heterogeneity within the AJCC stages and may help clinicians identify patients at high risk who require intensified follow‐up, ensuring the accuracy of patient counseling and facilitating the planning of personalized treatment. Finally, we do realize that favorable predictive accuracy is not necessarily related to usefulness in clinical practice. If the threshold probability of net benefits is unrealistic, the new prediction model may have limited applicability and may even be harmful compared with existing tools. As such, we conducted DCA in this study to confirm the clinical validity of these nomograms.

As the AJCC staging system is currently used system for assessment of prognosis of PDAC patients, we performed comparative analysis between the newly developed nomograms with this existing system. Based on the data, our nomograms showed better performance, with the higher c‐indexes and better AIC values, which were statistically different from those values in the AJCC staging system. In addition, DCA in this study indicated that the net benefit was consistently enhanced with the nomograms, suggesting that the nomograms are superior to the conventional AJCC staging system for the prediction and can be clinically useful. In general, the developed nomograms showed better stability and predictive ability.

Our study has several potential limitations that should be considered. First, serum carbohydrate antigen (CA) 19‐9 level, microRNA expression,^33^ surgical margin status, vascular invasion, and perioperative chemoradiotherapy were not integrated into the analysis, as this information was not available in the SEER dataset. Second, PDAC patients with distant metastases usually have poor prognosis, with the NCCN guidelines not recommending surgery for patients with M1 stage.^34^ In this retrospective study, all PDAC patients, including those with distant metastasis, underwent pancreatic surgery, such as pancreaticoduodenectomy, partial pancreatectomy, and total pancreatectomy. There is a possibility that distant metastasis was detected during the operation, and surgical removal of pancreatic tumors was performed to reduce the tumor load. The patients may then have been treated with adjuvant radiotherapy and chemotherapy after the operation. However, the SEER database did not specify the surgical procedure. As such, these might be the potential factors that could affect the prognosis of patients and make the c‐indexes of our nomograms mediocre. Third, owing to the competing risks, the accuracy for predicting OS tended to be lower in comparison with that for CSS in our study. Thus, Cox proportional hazard model with no competing risks for easier interpretation, comparison, and comprehension was chosen.^35^ Fourth, our nomograms aimed to select patients who might benefit from further care or additional interventions, such as strengthened treatments, adjuvant therapies, patient consulting, and intensified follow‐ups.

## 5 CONCLUSIONS

In this study, we have constructed and validated new nomograms for predicting the 1‐, 3‐, and 5‐year OS and CSS rates after surgical resection of PDAC. These nomograms showed good performance for the prediction of OS and CSS, which was superior to that of the existing, conventional AJCC staging system with an improved net benefit. Intrigued by the exciting findings in this retrospective study, we are planning to conduct a prospective clinical study, in which these nomograms will be evaluated for their performance in predicting survival among PDAC patients after surgery in our department. Therefore, these nomograms hold promise as novel prognosis models for improving the prediction of individualized postoperative survival and improving the care of PDAC patients following surgical resection.

## CONFLICT OF INTEREST

The authors declare that they have no conflict of interests.

## Data Availability

The datasets used and/or analyzed during this study are available from the corresponding author on reasonable request.
